# Whole exome sequencing reveals novel *COL4A3* and *COL4A4* mutations and resolves diagnosis in Chinese families with kidney disease

**DOI:** 10.1186/1471-2369-15-175

**Published:** 2014-11-07

**Authors:** Fujun Lin, Fan Bian, Jun Zou, Xiangru Wu, Jianping Shan, Wei Lu, Yao Yao, Gengru Jiang, Daniel Philip Gale

**Affiliations:** Department of Nephrology, Xin Hua Hospital, School of Medicine, Shanghai Jiao Tong University, Shanghai, China; UCL Centre for Nephrology, Royal Free Hospital, London, UK; Department of Pathology, Xin Hua Hospital, School of Medicine, Shanghai Jiao Tong University, Shanghai, China

**Keywords:** Collagen IV-related nephropathies, Whole exome sequencing, Novel mutation, Misdiagnosis

## Abstract

**Background:**

Collagen IV-related nephropathies, including thin basement membrane nephropathy and Alport Syndrome (AS), are caused by defects in the genes *COL4A3*, *COL4A4* and *COL4A5*. Diagnosis of these conditions can be hindered by variable penetrance and the presence of non-specific clinical or pathological features.

**Methods:**

Three families with unexplained inherited kidney disease were recruited from Shanghai, China. Whole exome sequencing (WES) was performed in the index case from each family and co-segregation of candidate pathogenic mutations was tested by Sanger sequencing.

**Results:**

We identified *COL4A4* missense variants [c.G2636A (p.Gly879Glu) and c.C4715T (p.Pro1572Leu)] in the 21-year-old male proband from family 1, who had been diagnosed with mesangial proliferative nephropathy at age 14. *COL4A4* c.G2636A, a novel variant, co-segregated with renal disease among maternal relatives. *COL4A4* c.C4715T has previously been associated with autosomal recessive AS and was inherited from his clinically unaffected father. In family 2, a novel *COL4A3* missense mutation c.G2290A (p.Gly997Glu) was identified in a 45-year-old male diagnosed with focal segmental glomerulosclerosis and was present in all his affected family members, who exhibited disease ranging from isolated microscopic hematuria to end stage renal disease (ESRD). In family 3, ESRD occurred in both male and females who were found to harbor a known AS-causing *COL4A5* donor splice site mutation (c.687 + 1G > A). None of these variants were detected among 100 healthy Chinese individuals.

**Conclusion:**

WES identified 2 novel and 2 known pathogenic *COL4A3*/*COL4A4*/*COL4A5* mutations in 3 families with previously unexplained inherited kidney disease. These findings highlight the clinical range of collagen IV-related nephropathies and resolved diagnostic confusion arising from atypical or incomplete clinical/histological findings, allowing appropriate counselling and treatment advice to be given.

**Electronic supplementary material:**

The online version of this article (doi:10.1186/1471-2369-15-175) contains supplementary material, which is available to authorized users.

## Background

The type IV collagen α3α4α5 chain is a major component of the glomerular basement membrane (GBM) and is a heterotrimer that is encoded by three genes: *COL4A3*, *COL4A4* and *COL4A5.* While *COL4A3* and *COL4A4* are located on chromosome 2, *COL4A5* lies on the X chromosome. Mutations in any of these genes can lead to type IV collagen-related nephropathy in which there is disruption of the normal glomerular basement membrane architecture and kidney disease [1]. In general, heterozygous mutations result in mild disease characterized by focal or diffuse thinning of the GBM, with or without isolated microscopic hematuria (MH) and termed thin basement membrane nephropathy (TBMN). In a minority (estimated at 10-20%) of patients with TBMN and heterozygous mutation of *COL4A3*, *4* or *5* there is progressive renal dysfunction with end stage renal disease (ESRD) in later life, usually after the fifth decade [2]. Males hemizygous for a *COL4A5* mutation, and individuals of either sex with homozygous or compound heterozygous *COL4A3/4* mutations, are at risk of X-linked and autosomal recessive Alport Syndrome (AS) respectively, in which there is a high likely of ESRD within the first three decades of life, associated with sensorineural deafness and ocular abnormalities, including asymptomatic dot and fleck retinopathy and lenticonus.

Genetic testing is the gold standard in diagnosing collagen IV-related nephropathies. In AS (a rare disease with 1:5000 prevalence), X-linked (XL) inheritance is reported in 80-85% of patients autosomal recessive AS (ARAS) accounts for 15% of cases. TBMN reported to affect at least 1% of the worldwide population is frequently associated with heterozygous mutations of *COL4A3*, *COL4A4* or *COL4A5*. Although more than 1000 mutations (nearly 300 in *COL4A3* and *COL4A4*, and 756 in *COL4A5*) have been identified in AS and TBMN [3], the challenges in the diagnosis of collagen IV nephropathies remain. The large size of *COL4A3*, *COL4A4* and *COL4A5* genes, comprising 48–53 exons each, and the lack of mutational hot spots make individual screening of multiple genes by Sanger sequencing difficult and expensive. While historically a *COL4A5* mutation has been identified in 90% of XLAS [4], in suspected autosomal disease the detection rate of detection of *COL4A3/COL4A4* mutations has been reported as low as 20% [5]. The clinical and light microscopic (LM) kidney biopsy findings of some collagen IV-related nephropathy patients are difficult to distinguish from other conditions such as focal segmental glomerulosclerosis (FSGS) and glomerulonephritis, which may lead to incorrect diagnosis [6]. In families presenting with isolated microscopic hematuria only, kidney biopsy may not be clinically indicated and genetic testing is frequently not performed, presumably owing to the cost.

Recent availability of next-generation sequencing (NGS) technologies including whole exome sequencing (WES) make it possible to simultaneously test thousands of genes in an unbiased, high-throughput and cost-efficient manner. WES has recently corrected the diagnosis of AS patients mistaken for familial focal segmental glomerulosclerosis (FSGS) [7, 8]. Targeted exome capture of *COL4A3*/*COL4A4*/*COL4A5* followed by NGS has also been used clinically to screen clinically suspected AS patients [9, 10]. In this study using WES, we identified novel and rare type IV collagen gene mutations in 3 unrelated Chinese families with glomerulonephritis and no extra-renal involvement, in whom diagnosis had been difficult to make because of atypical clinical presentation and complex or incomplete histological findings. Since affected members of all 3 families had progressed to ESRD, precise molecular diagnosis was clinically valuable because it allowed genetic counseling and predictive testing to be offered to relatives, aided therapeutic decision-making and allowed more accurate prognostic advice to be given.

## Methods

### Human patients

The study was conducted according to protocols approved by the Ethical Committee of Shanghai Xin Hua Hospital (No. XHEC-D-2014-002), and written informed consent was obtained from each participant. DNA was extracted from blood samples using QIAamp DNA midi kit (Qiagen). DNAs from 100 ethnically matched Chinese healthy individuals were used as controls.

### Whole exome sequencing (WES) analysis

Targeted exome capture was performed using genomic DNA from each subject using SureSelect Human All Exon Target Enrichment System (Agilent). Paired-end sequencing (76 bp) was performed using the Genome Analyzer IIx (Illumina Inc). Read alignment to the human genome assembly hg19 using the novoalign alignment tool (Novocraft Technologies). Variant calling and annotations were obtained using the Samtools mpileup utility [11] and Annovar tools [12] respectively.

To identify pathogenic variants, a stepwise filtering process was used that firstly removed non-coding, synonymous and common variants (minor allele frequency, MAF >0.005 in the 1000 genome project database or our in-house database of >800 exomes). The remaining non-synonymous coding and splice-site variants were further filtered to include those occurring in a set of 32 genes (Additional file 1: Table S1) known to be associated with non-syndromic familial nephropathy and/or kidney disease with phenotypes compatible with those in the 3 families. The 32 genes were selected after search in databases including PubMed, the Online Mendelian Inheritance in Man (OMIM) and the Human Gene Mutation Database (HGMD). Mapping, coverage and filtering statistics are summarized in Additional file 2: Table S2 and Additional file 3: Table S3.

Multiple protein sequence alignment to assess evolutionary conservation of residues was performed using UCSC Genome Browser (http://genome.ucsc.edu).

### Confirmation of candidate variants using Sanger sequencing

Candidate variants identified by WES were validated by Sanger sequencing using an ABI PRISM 3730xl Genetic Analyzer (Applied Biosytems). Primers sequences are available upon request. Co-segregation of candidate variants was tested in all family members for whom DNA was available and co-segregating candidate variants were genotyped in 100 unrelated Chinese healthy controls.

## Results

### Clinical description of the 3 families and whole exome sequencing results

#### Family 1

**Figure 1:**
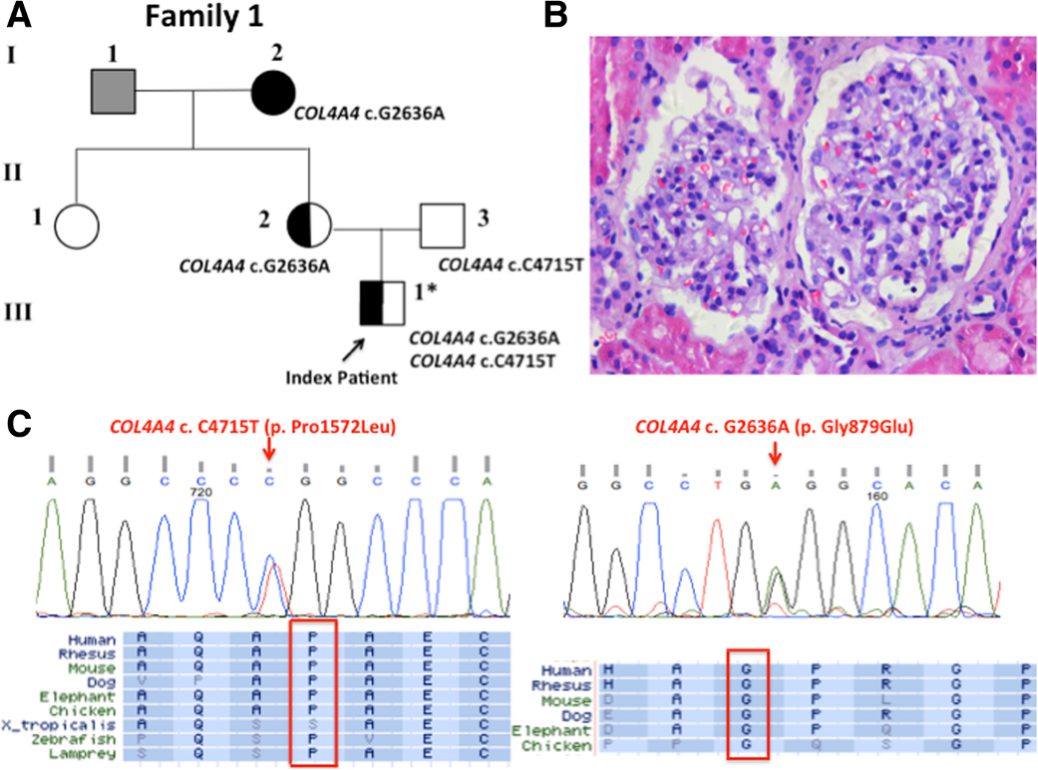
***COL4A4*** **mutations identified in Family 1. (A)** Pedigree for family 1. A full-shaded icon denotes ESRD; individuals with microscopic hematuria and proteinuria but with normal renal function are indicated with a half-shaded icon; individual with unknown phenotype is indicated in gray. Asterisk indicates the individual examined by whole exome sequencing. **(B)** Light microscopic renal biopsy from III-1, stained with hematoxylin and eosin showing segmental mesangial cell proliferation (X40). **(C)** Sanger sequencing electropherograms confirming the heterozygous missense *COL4A4* c.G2636A (p.Gly879Glu) and c.C4715T (p.Pro1572Leu) mutations, and multiple species protein sequence alignment showing conservation of the mutated Gly879 and Pro1572 residues.

The index case (III-1) was a 21 year-old male who presented with microscopic hematuria (MH) and proteinuria (1.0 g/24 hrs) at age 14 (Figure 1A). Renal biopsy at that time was inconclusive: LM showed mesangial proliferative nephropathy (MsPGN) (Figure 1B) with segmental afferent arteriolar C3 deposition (1+) on immunofluorescence (IF). EM revealed early segmental obliteration in one capillary loop and mesangial cell proliferation with no apparent GBM abnormalities. After treatment with angiotensin converting enzyme inhibitor (ACEi) and oral corticosteroids, proteinuria decreased to 300-500 mg/24 hrs but MH persisted, and renal function remained normal. His grandmother (I-2) had undergone a kidney biopsy at age 55 and this was reported as showing typical LM and IF findings of IgA nephropathy. She had been treated with oral corticosteroids for 5 years but progressed to ESRD 8 years later. III-1’s mother (II-2) also developed MH and proteinuria in her 30s but declined renal biopsy.

We performed WES in individual III-1 and this identified two heterozygous candidate *COL4A4* missense mutations, c.G2636A (p.Gly879Glu) and c.C4715T (p.Pro1572Leu). Sanger sequencing (Figure 1C) confirmed the presence of the *COL4A4* c.G2636A in II-2 and I-2 and revealed that the *COL4A4* c.C4715T was inherited from III-1’s father (II-3) who is well, with normal urinalysis and blood pressure. The novel *COL4A4* c.G2636A mutation predicts a glycine to glutamine substitution in a collagenous domain of the protein. Further Sanger sequencing confirmed this mutation not present in 100 healthy ethnicity-matched controls. *COL4A4* c.C4715T mutation predicts a proline to leucine substitution in the non-collagenous (NC1) domain of the protein. Unlike collagenous domain glycine substitutions, *COL4A4* point mutations resulting in a substitution of proline for another amino acid are rarely associated with disease: of the 15 unique *COL4A4* missense mutations involving proline that are present in the LOVD database (http://grenada.lumc.nl/LOVD2/COL4A/), only this and one other, p.Pro1132Leu, are considered to be likely pathogenic [13]. However, several pathogenic proline substitutions have been reported in *COL4A5* (e.g. p.Pro628Leu [14]; p.Pro739Ser [15]; p.Pro1523Thr [16]; p.Pro1590Leu [17]), establishing that this type of mutation, occurring in either collagenous or non-collagenous domains of the protein can disrupt Type IV collagen. Since the *COL4A4* p.Pro1572Leu mutation lies in the non-collagenous domain at a residue that is highly conserved across all human Type IV collagen α chains and has previously been described in two separate ARAS cases [18, 19], we believe there is a high likelihood it is a pathogenic variant. Using PolyPhen 2 (http://genetics.bwh.harvard.edu/pph2/) and SIFT (http://sift.jcvi.org) to predict possible functional effects, both *COL4A4* c.G2636A (p.Gly879Glu) and c.C4715T (p.Pro1572Leu) were classified as pathogenic and are highly conserved across multiple organisms (results summarized in Table 1).

**Table 1:** **Pathogenic variants identified by whole exome sequencing**

Family	Samples analyzed by WES	Pathological diagnosis before WES analysis	Pathogenic variants	Zygosity	Novel or clinical	SIFT ^a^	PolyPhen2 ^b^	Mutation at conserved position
1	III-1	MsPGN	*COL4A4* c.G2636A (p.Gly879Glu)	Het	Novel	0.001	1.0	Yes
			*COL4A4* c.C4715T (p. Pro1572Leu)	Het	Clinical [18, 19]	0	1.0	Yes
2	II-4	FSGS	*COL4A3* c.G2290A (p.Gly997Glu)	Het	Novel	0	1.0	Yes
3	II-2	Biopsy not performed	*COL4A5* (c.687 + 1G > A)	Het	Clinical [20]	N/A	N/A	Yes

^a^A SIFT score of <0.05 is predicted to be deleterious.

^b^A Polyphen2 score is predicted to be “probably damaging” if it is >0.85, “possibly damaging” if between 0.85 and 0.2, and “benign” if < 0.2.

MsPGN, mesangial proliferative nephropathy; FSGS, focal segmental glomerulosclerosis; N/A, not available.

**Figure 2:**
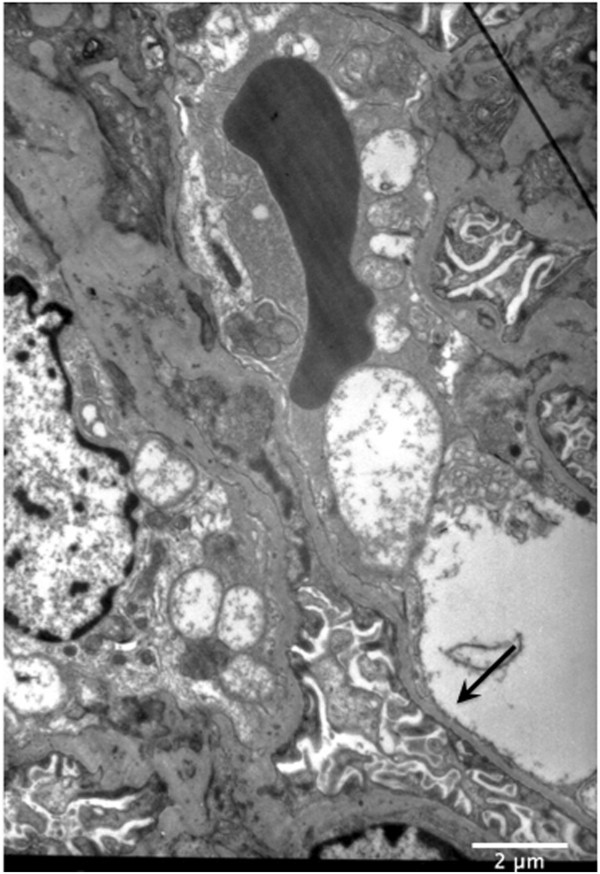
**Reprocessed electron micrographs of the renal biopsy from patient III-1 of family 1.** Capillary loop showing segmental thinning of GBM (arrow) with no irregular GBM thickening and lamellation (X 4000).

Given the unexpected compound heterozygous *COL4A4* mutations identified in III-1, we reprocessed and reanalyzed his EM specimen. Only segmental thinning of GBM was found with no irregular GBM thickening and lamellation (Figure 2), consistent with a Type IV collagen-related disorder, but not diagnostic of recessive AS. Subsequent visual and hearing examinations were normal. His grandmother’s (I-2) renal biopsy, performed >15 years ago was unavailable for review. The presence of two *in trans* mutations in III-1 may explain why he has developed proteinuria early and suggests an increased risk of renal impairment developing at younger age in him compared with his heterozygous relatives.

#### Family 2

The index patient (II-4) from Family 2 was a 45-year-old Chinese male with over 10 years’ history of proteinuria (2-3 g/24 hr), MH, hypertension and recurrent gout attacks. Renal biopsy was performed at age 39 with the diagnosis of FSGS on LM. EM was not performed because of sample insufficiency. He was treated with corticosteroids for 2 years but progressed to stage 4 chronic kidney disease (GFR = 25 ml/min by age 45). His mother (I-2) had 20 years’ history of proteinuria and MH and progressed to ESRD aged 55. Sisters (II-2 and II-3) of the index patient both exhibited MH and proteinuria (0.5-1.5 g/24 hr) with preserved renal function, and II-4’s niece (III-1) was found to have isolated MH during her pregnancy. None of the affected family members reported hearing or visual impairment.

**Figure 3:**
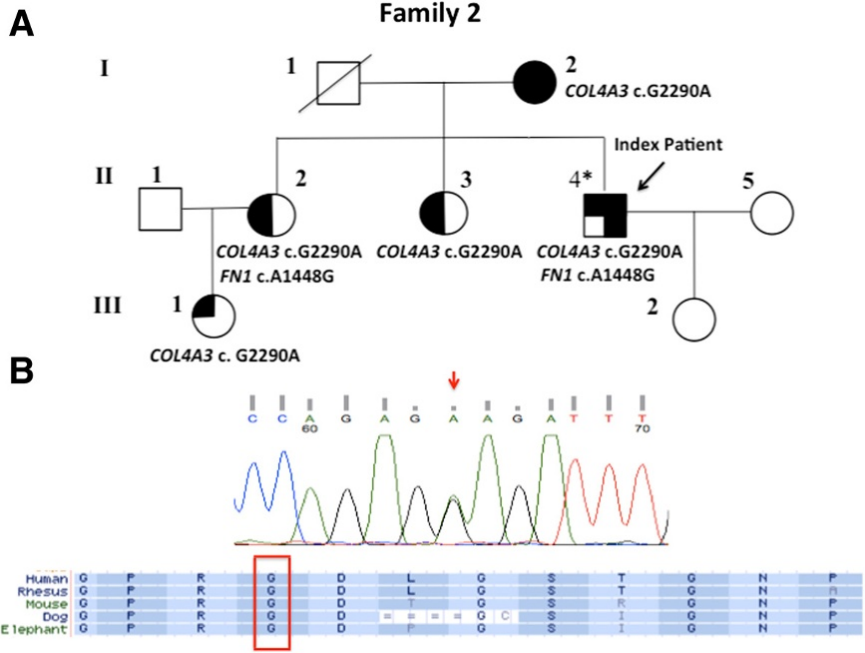
***COL4A3*** **mutation identified in Family 2. (A)** Pedigree for family 2. A full-shaded icon denotes ESRD; A 3/4-shaded icon denotes individual with impaired renal function; a half-shaded icon denotes individuals with microscopic hematuria and proteinuria but with normal renal function; 1/4-shaded icon denotes isolated hematuria. Asterisk indicates the individual examined by whole exome sequencing. **(B)** Sanger sequencing electropherograms confirming the heterozygous missense *COL4A3* c.G2290A (p.Gly997Glu) mutation, and multiple species protein sequence alignment showing conservation of the mutated Gly997 residue.

WES was performed on II-4. This identified a novel heterozygous *COL4A3* missense mutation [c.G2290A (p.Gly997Glu)] and a rare heterozygous *FN1* missense mutation [c.A1448G (p.Glu483Arg)] with MAF of 0.0005. No non-synonymous rare variants were identified in genes associated with inherited FSGS. Sanger sequencing confirmed the cosegregation of *COL4A3* c.G2290A in all 5 affected family members but the *FN1* missense mutation [c.A1448G (p.Glu483Arg)] was not present in I-2, II-3 and III-1 (Figure 3A). *COL4A3* c.G2290A mutation predicts substitution of a highly conserved glycine by glutamine in the collagenous domain of the protein (Figure 3B) and was classified as pathogenic by *in silico* software (Results summarized in Table 1). This mutation was not detected among 100 unrelated Chinese control individuals.

#### Family 3

The index patient (II-2) in kindred 3 was a 65 year-old female who developed nephrotic syndrome at age 30, and renal biopsy was not performed at that time. There was no response to oral corticosteroids given for 3 months. She progressed to ESRD at age 57. Her mother (I-2) and one of her younger sisters (II-5) reached ESRD at ages 40 and 50 respectively, and her younger brother (II-4) received a kidney transplant at age 32 and died 8 years later. II-5’s son (III-2) developed heavy proteinuria and edema at age 2 and progressed to CKD3 (GFR of 55 ml/min) at age 30. The other sister (II-3) of the index patient developed proteinuria with increased serum creatinine (150 umol/L) at age 50, with no MH. Her renal function remained stable on no medical therapy over the next 10 years. Ultrasound in II-3 showed small kidneys with no cysts. II-3 and III-2 declined renal biopsy. None of the affected family members reported hearing or visual impairment.

**Figure 4:**
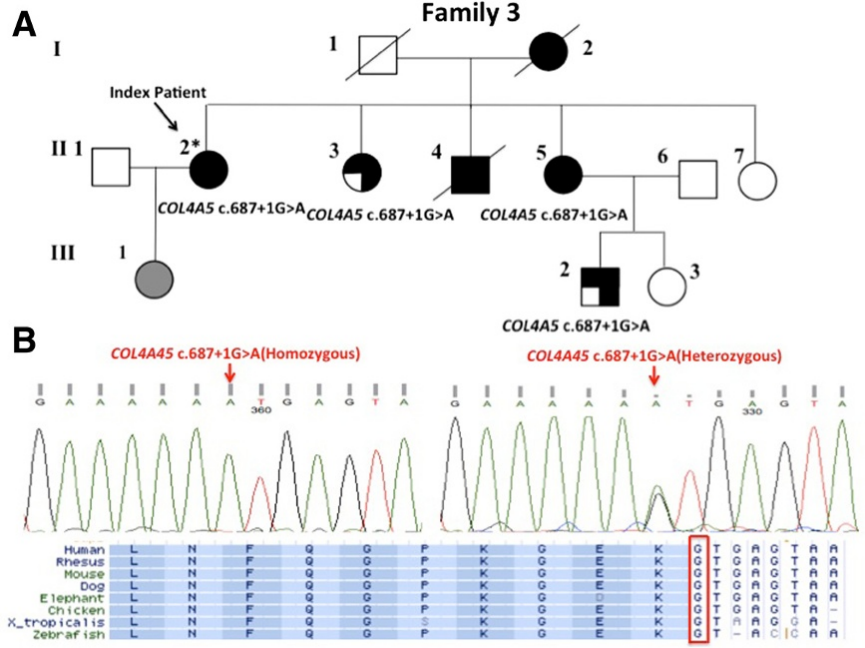
**Overview of the** ***COL4A5*** **mutation identified in Family 3. (A)** Pedigree for family 3. A full-shaded icon denotes ESRD; a 3/4-shaded icon denotes impaired renal function; individual with unknown phenotype is indicated in gray. Asterisk indicates the individual examined by whole exome sequencing. **(B)** Sanger sequencing electropherograms confirming the *COL4A5* donor splice site mutation (c.687 + 1G > A), and multiple species sequence alignment showing conserved GT in the donor splice site.

Whole exome sequencing was performed in II-2. After variant filtering, we identified a *COL4A5* donor splice site variant (c.687 + 1G > A), which was previously reported to cause XLAS [20], and a rare heterozygous *CUBN* missense mutation [c.C9206T (p.Thr3069Ile)] with MAF of 0.003 (Figure 4A). Sanger sequencing (Figure 4B) confirmed that *COL4A5* (c.687 + 1G > A) cosegregated in all affected members in this family while the *CUBN* variant was not present in affected individuals II-3 and III-2, and was present in clinically unaffected individuals II-7 and III-3 (results summarized in Table 1). Sanger sequencing confirmed that *COL4A5* (c.687 + 1G > A) was not present in 100 healthy Chinese controls.

## Discussion

Current options for genetic testing include Sanger sequencing of candidate gene(s), next generation sequencing of a selected panel of candidate genes, WES, and whole genome sequencing. In the clinical setting, targeted panels are gaining favor because of the relatively modest cost and the detail with which the targeted regions are analyzed which can allow reliable copy number estimation, detecting or excluding within-gene duplications and deletions. Two recent studies using targeted panels of *COL4A3/A4/A5* for the genetic diagnosis in patients suspected of having a Type IV collagen-related nephropathy identified a likely pathogenic mutation in 55% and 83.2% patients tested [9, 10]. A major limitation of this approach is that if the panel used does not include the gene responsible in a patient, the mutation will not be detected. This is a significant problem in the current era when new genes are being implicated in Mendelian kidney diseases each year. WES yields data on a far greater number of genes, including those not initially considered candidates for the phenotype being investigated. This allows a diagnosis to be made where detailed phenotype data (such as a kidney biopsy) are misleading or lacking, but may detect variants that predict disease unrelated to the indication for testing in an individual patient, and which they would rather not know about. These considerations must be taken into account when consenting for and analyzing WES data, and are of course avoided when only genes known to be related to the trait under investigation are sequenced. A further advantage is that unbiased screening of large numbers of genes may allow epistatic effects to be detected, such as that of *NPHS2* variant p.R229Q which, although not detected in any of the patients in this study, is known to be associated with proteinuria and renal impairment in TBMN [21, 22]. Whole genome sequencing is currently less widely applied outside a research setting – the amount of sequencing, data storage and analysis required are significantly greater than for WES and it is not clear if the associated costs are justified when the vast majority of detectable disease-causing variants lie within coding regions that are covered by WES.

The 3 families in this study posed well-recognized diagnostic challenges in kidney disease, including atypical or late clinical presentation with unavailable or inconclusive pathology findings: patients from family 1 and family 2 in this study were initially diagnosed with MsPGN, IgAN and FSGS. Some individuals were treated with extended courses of corticosteroids that very likely had no beneficial effect on their renal disease and would not have been used had the correct underlying diagnosis been known to the treating clinician at the time.

In family 1, although we identified compound heterozygous *COL4A4* mutations in the index patient, his reprocessed EM findings and clinical course of disease was more suggestive of heterozygous *COL4A*-related disease than ARAS. Interestingly, the index patient’s grandmother was initially diagnosed with IgAN and the unexpected identification of the co-segregating *COL4A4* [c.G2636A (p.Gly879Glu)] mutation in her suggested co-existence of a collagen IV-related nephropathy. Since she was the only family member who developed ESRD in this family, her poor renal outcome may be attributable to the coincidence of these two kidney diseases, although it is well recognized that a single heterozygous *COL4A3/COL4A4* mutation is associated with late-onset ESRD in up to 20% of patients [2]. Superimposition of other comorbidities, such as IgAN, in collagen IV-related nephropathy (especially in TBMN) is not rare. A previous study has shown that 30% of familial IgAN cases had GBM abnormalities and TBMN could be a significant contributor to disease in these families [23].

LM findings of AS often resemble those of FSGS or MsPGN. Data retrieved from the Alport Syndrome database in South China recently showed that the misdiagnosis rate of AS is 13% (52 out of 398), with MsPGN (26.9%) and FSGS (19.2%) being the most common misdiagnoses [24]. In family 2, WES identified no known or likely FSGS-associated variants, but a novel heterozygous *COL4A3* [c.G2290A (p.Gly997Glu)] mutation that is predicted to have a functional impact on the collagen IV heterotrimer structure. This strongly suggests an underlying diagnosis of collagen IV-related nephropathy rather than an inherited or acquired podocytopathy.

Apart from pathological heterogeneity, collagen IV-related nephropathy is clinically heterogeneous within and between families. In male XLAS patients, the mutation location and nature predict clinical severity. Large deletions and insertions, rearrangements, splicing and nonsense mutations, and mutations within the NC1 domain of *COL4A5* are associated with severe consequences with ESRD occurred before age 20, whereas missense mutations involving the collagenous domain are responsible for a less severe type of AS [25]. In female XLAS patients, the explanation for the wide variability in outcomes is uncertain, as genotype-phenotype correlation has not been observed. The largest study, of 288 female patients from 195 families, showed that 18% patients progressed to ESRD over the course of the follow-up and only 12% of them reached ESRD by age 40 [26]. In family 3, in which we detected a *COL4A5* donor splice site mutation (c.687 + 1G > A) known to cause XLAS, the disease was unusually severe (for X-linked disease) in the female patients I-2, II-2 and II-5 and unusually mild in the male patient III-2. The intrafamilial heterogeneity in female patients of family 3 may be due to variable X-chromosomal inactivation [27]. Other factors such as unknown modifying genes and environmental factors may also affect the natural history of disease in different affected family members.

Recently, the first clinical application of WES reported that the success rate of mutation identification in patients with genetic disorder is approximately 25% [28]. In our study, sequencing one affected family member using WES, combined with stepwise variant filtering strategy, we identified mutations of collagen IV genes in 3 undiagnosed glomerulonephritis families with no extra-renal involvement. This result shows that WES is a powerful diagnostic tool that can complement renal biopsy and can non-invasively provide a diagnosis in patients with familial kidney disease, particularly when clinical information is limited or non-specific.

## Conclusion

WES identified 2 novel and 2 known pathogenic *COL4A3*/*COL4A4*/*COL4A5* mutations in 3 families with previously unexplained inherited kidney disease. These findings highlight the clinical range of collagen IV-related nephropathies and resolved diagnostic confusion arising from atypical or incomplete clinical/histological findings, allowing appropriate counselling and treatment advice to be given.

## Electronic supplementary material

Additional file 1: Table S1: 32 genes known to be associated with non-syndromic familial nephropathy and/or kidney disease with phenotypes compatible with those in the 3 families. (DOCX 18 KB)

Additional file 2: Table S2: Summary statistics for exome sequencing-mapping and coverage. (DOCX 15 KB)

Additional file 3: Table S3: Variant filtering statistics. (DOCX 14 KB)
