# Supplementary material for: Whole exome sequencing reveals novel COL4A3 and COL4A4 mutations and resolves diagnosis in Chinese families with kidney disease
Source: BMC Nephrol. 2014 Nov 7;15:175. doi: 10.1186/1471-2369-15-175 (PMC4233041; doi:10.1186/1471-2369-15-175)
Supplement: Supplementary file 1 — Additional file 1: Table S1: 32 genes known to be associated with non-syndromic familial nephropathy and/or kidney disease with phenotypes compatible with those in the 3 families. (DOCX 18 KB) [file 12882_2014_864_MOESM1_ESM.docx]

**Supplementary Table S1. 32 genes known to be associated with non-syndromic familial nephropathy and/or kidney disease with phenotypes compatible with those in the 3 families**

| **Gene** | **Protein** | **Inheritance pattern** | **Disease association** |  |
| --- | --- | --- | --- | --- |
| *ACTN4* | α-Actinin 4 | AD | FSGS |  |
| *ADCK4* | AARF domain-containing kinase 4 | AR | SRNS |  |
| *ANLN* | Anillin | AD | FSGS |  |
| *ARHGDIA* | Rho GDP-dissociation inhibitor α | AR | NS |  |
| *ARHGAP24* | Rho GTPase-activating protein 24 | AD | FSGS |  |
| *CD2AP* | CD2-associated protein | AD | FSGS/SRNS |  |
| *CFHR5* | Complement factor H-related protein 5 | AD | C3 glomerulopathy |  |
| *COL4A3* | Collagen, type IV, α3 | AD/AR | Alport's disease |  |
| *COL4A4* | Collagen, type IV, α4 | AD/AR | Alport's disease |  |
| *COL4A5* | Collagen, type IV, α5 | XL | Alport's disease |  |
| *COQ2* | Coenzyme Q2 homolog | AR | Mitochondrial disease/isolated nephropathy | |
| *CUBN* | Cubilin | AR | NS |  |
| *DGKE* | Diacylglycerol kinase ε | AR | NS/aHUS |  |
| *DSTYK* | Dual serine/theronine and tyrosine protein kinase | AD | CAKUT |  |
| *FN1* | Fibronectin 1 | AD | Familial glomerulopathy with fibronectin deposits | |
| *HNF1B* | HNF1 homobox B | AD | Autosomal dominant tubulointerstitial kidney disase | |
| *INF2* | Inverted Formin-2 | AD | FSGS |  |
| *LAMB2* | Laminin-β2 | AR | DMS/FSGS |  |
| *LMX1B* | LIM homeobox transcription factor 1β | AD | Nail petella syndrome/isolated FSGS | |
| *MYH9* | Nonmuscel myosin heavy chain 9 | AD | MHY9-related disease | |
| *MYO1E* | Myosin IE | AD | FSGS |  |
| *NPHS1* | Nephrin | AR | SRNS |  |
| *NPHS2* | Podocin | AR | SRNS |  |
| *PAX2* | Paired box gene 2 | AD | FSGS/CAKUT/papillorenal syndrome(PRS) | |
| *POXDL* | Podocalyxin | AD | FSGS |  |
| *PLCE1* | Phospholipase C, ε1 | AR | DMS/FSGS |  |
| *PTPRO* | Protein tyrosine phosphatase, receptor type, O | AR | FSGS |  |
| *REN* | Renin | AD | Autosomal dominant tubulointerstitial kidney disase | |
| *TTC21B* | IFT139 | AR | FSGS/NPHP |  |
| *TRPC6* | Transient receptor potential cation channel, subfamily C, member 6 | AD | FSGS |  |
| *UMOD* | Uromodulin | AD | Autosomal dominant tubulointerstitial kidney disase | |
| *WT1* | Wilms tumor 1 | AD | Frasier syndrome or /FSGS | |
