# Supplementary material for: Whole exome sequencing reveals novel COL4A3 and COL4A4 mutations and resolves diagnosis in Chinese families with kidney disease
Source: BMC Nephrol. 2014 Nov 7;15:175. doi: 10.1186/1471-2369-15-175 (PMC4233041; doi:10.1186/1471-2369-15-175)
Supplement: Supplementary file 2 — Additional file 2: Table S2: Summary statistics for exome sequencing-mapping and coverage. (DOCX 15 KB) [file 12882_2014_864_MOESM2_ESM.docx]

**Supplementary table 2. Summary statistics for exome sequencing-mapping and coverage**

|  | **III-1 (Family 1)** | **II-4 (Family 2)** | **II-2 (Family 3)** |
| --- | --- | --- | --- |
| total_reads | 72468757 | 71271554 | 96181581 |
| mapped_to_target_reads | 52939162 | 52217263 | 67824633 |
| Percentage | 73.05 | 73.27 | 70.52 |
| mapped_to_target_reads_plus_150bp | 59360736 | 58259863 | 75648843 |
| Percentage | 81.91 | 81.74 | 78.65 |
| mean_coverage | 98.89 | 97.62 | 127.07 |
| accessible Target_bases | 33323618 | 33323618 | 33323618 |
| accessible Target_bases_1x | 32687047 | 32665442 | 32963095 |
| Percentage | 98.09 | 98.03 | 98.92 |
| Target_bases_5x | 32083508 | 32033300 | 32647248 |
| Percentage | 96.28 | 96.13 | 97.97 |
| Target_bases_10x | 31345444 | 31277432 | 32369083 |
| Percentage | 94.06 | 93.86 | 97.14 |
| Target_bases_20x | 29400711 | 29325587 | 31648086 |
| Percentage | 88.23 | 88 | 94.97 |
