# Supplementary material for: Whole exome sequencing reveals novel COL4A3 and COL4A4 mutations and resolves diagnosis in Chinese families with kidney disease
Source: BMC Nephrol. 2014 Nov 7;15:175. doi: 10.1186/1471-2369-15-175 (PMC4233041; doi:10.1186/1471-2369-15-175)
Supplement: Supplementary file 3 — Additional file 3: Table S3: Variant filtering statistics. (DOCX 14 KB) [file 12882_2014_864_MOESM3_ESM.docx]

**Supplementary table 3. Variant filtering statistics**

|  | **III-1 (family 1)** | **II-4 (family 2)** | **II-2 (family 3)** |
| --- | --- | --- | --- |
| **Total variants** | 24,617 | 24,586 | 25,554 |
| **After exclusion of non-coding and synonymous variants** | 13,605 | 13,654 | 14,205 |
| **After filtering common variants** | 603 | 660 | 643 |
| **Remaining variants within candidate genes** | *COL4A4* c.G2636A; COL4A4 c.C4715T | *COL4A3* c.G2290A;  *FN1* c.A1448G | *COL4A45* (c.687+1G>A); *CUBN* c.C9206T |
